# Analysis of MTHFR C677T genotype and related factors in H-type hypertension in Tibet, China

**DOI:** 10.1186/s12872-024-04015-6

**Published:** 2024-07-08

**Authors:** Jiaojiao Yan, Yufei Zhang, Yaxi Zhou, Yang Wan, Hai Xiong

**Affiliations:** 1 Department of Neurology, West China School of Public Health and West China Fourth Hospital, Sichuan University, No.17, Section 3, Renmin South Road, Wuhou District, Chengdu, Sichuan 610041 China; 2 Medical College, Tibet University, No.10, Zangda East Road, Chengguan District, Lhasa, Tibet Autonomous Region 850000 China

**Keywords:** Tibetans, H-type hypertension, Hyperhomocysteinemia, MTHFR C677T gene

## Abstract

**Background and aims:**

H-type hypertension is essential hypertension combined with high homocysteine, and both synergistically increase the risk of cardiovascular and cerebrovascular events. The aim of this study was to investigate the risk factors of H-type hypertension in Tibetan plateau population and correlation with MTHFR C677T gene.

**Methods and results:**

A multi-stage cluster random sampling method was used to select the research subjects in Tibet Autonomous Region from June 2020 to November 2021. Among Tibetans, the incidence of H-type hypertension accounted for 84.31% of hypertensive patients. The logistic regression analysis demonstrated that age, uric acid (UA), triglyceride (TG) and low-density lipoprotein cholesterol (LDL-C) were risk factors for the prevalence of H-type hypertension, the OR (95% CI) was 1.083(1.073–1.094), 1.002(1.001–1.004), 1.240(1.050–1.464) and 2.274(1.432–3.611), respectively. MTHFR C677T TT genotype patients with H-type hypertension OR (95% CI) was 1.629(1.004–2.643). Based on this, a nomogram model was established, and the reliability of the model was proved by area under ROC curve, Brier score and average absolute error. The model’s results indicate that for every five years of age, the score increases by 6 points; for a 2mmol/L increase in TG, the score increases by 5.5 points; for a 1mmol/L increase in LDL-C, the score increases by 10 points; and individuals with the TT genotype receive 8 points. The higher the score, the greater the risk of disease.

**Conclusion:**

The MTHFR C677T TT genotype is a risk locus for Tibetan patients with H-type hypertension, with age, TG, and LDL-C were identified as risk factors for the disease.

## Introduction

In the Chinese population, the prevalence of H-type hypertension was found to be 26.9% in the total population, including 81.8% of men and 62.8% of women, and 73.1% in patients with hypertension [1]. Hyperhomocysteinemia may be a potential independent risk factor for cardiovascular disease, dementia, and other related disorders [2], and a degree of synergy between hypertension and hyperhomocysteinemia can lead to an increased burden on the cardiovascular system. This suggests that the coexistence of hypertension and hyperhomocysteinemia may exacerbate the overall risk and impact of these conditions on individuals’ health. Studies have estimated that the incidence of adverse cardiovascular events in patients with H-type hypertension is approximately five times higher than in patients with essential hypertension alone and approximately 25–30 times higher than in the healthy population [3] 0.5,10-methylenetetrahydrofolate reductase (MTHFR) plays an important role as a key enzyme in folate metabolism and plasma Hcy remethylation in the body, and the C677T (rs1801133) locus gene of MTHFR is the most common one that can lead to reduced or diminished activity of the MTHFR enzyme and subsequent elevation of blood Hcy levels [4, 5]. The C to T substitution at nucleotide 677 site leads to the substitution of valine codon for alanine, causing a decrease in the activity of MTHFR enzyme, which inhibits the remethylation of Hcy to methionine and affects the distribution of folate, leading to the accumulation of Hcy in the bloodstream in the presence of an impaired folate state [6–8].

There are significant geographic and ethnic differences in the prevalence of the MTHFR C677T gene frequency in China, and there are associations with a variety of diseases that affect the disease characteristics of certain populations [9]. However, they should not be viewed a priori as genetic defects that lead to disease, and there is a need for region- and/or race-specific gene-disease association databases to further validate whether susceptibility loci do indeed exist between diseases. [4]. The intricate interplay between the biochemical actions of MTHFR and its genetic variants highlights the importance of understanding these mechanisms in the context of diseases such as H-type hypertension [10].

Meta-analysis found that MTHFR C677T gene polymorphism was associated with cardiovascular disease, tumor, neurological disorders, and adverse pregnancy outcomes [11–14]. In addition, it is noteworthy that MTHFR C677T gene polymorphisms exhibit intergroup, regional, and age-related variations, potentially contributing to heterogeneous susceptibilities to diseases across diverse populations [15]. The aim of our study was to understand the genetic characteristics of the MTHFR C677T gene in Tibetan patients with H-type hypertension in the Tibetan population in the Tibetan region, and to clarify the relevant factors affecting the occurrence of H-type hypertension. To propose individualized prevention and treatment of H-type hypertension in response to the findings and to decrease the prevalence of cardiovascular and cerebrovascular diseases in the region.

## Methods

### Design and study participants

During the period from June 2020 to November 2021, a multi-stage whole-group random sampling approach was used to select participants. Using a strict randomization process, 3 cities were first randomly selected from the 7 cities in the autonomous region of Tibet; 1–3 counties were randomly selected from each city; 2 townships were selected from each county; and 2–3 neighbourhood or village committees were randomly selected from each township. A total of 2 313 people were included based on actual participation and valid questions and answers. The subjects were categorized into 4 distinct groups based on their blood pressure and plasma Hcy levels: H-type hypertension group, essential hypertension group, isolated Hyperhomocysteinemia group, and normal control group. Inclusion criteria:①individuals aged ≥ 18 years old;②Tibetan residents who had not relocated from their essential place of residence for a continuous period of 6 months preceding the data collection phase. Exclusion criteria:①individuals suffering from mental illness, serious cardiovascular and cerebrovascular diseases, liver and kidney failure, infectious diseases, autoimmune diseases and other significant medical conditions;②individuals who cannot cooperate to complete the survey;③individuals with incomplete data;④pregnant and breastfeeding women. The study participants provided voluntarily signed the informed consent form, and the research protocol obtained approval from the Ethics Committee of Tibet University.

### Clinical information

According to “the 2018 Chinese Guidelines for the Prevention and Treatment of Hypertension” [16], Hyperhomocysteinemia is defined as a plasma Hcy level ≥ 15 µmol/L. Hypertension is defined as three on-site blood pressure measurements without antihypertensive medication and averaged, with systolic blood pressure (SBP) ≥ 140 mmHg and/or diastolic blood pressure (DBP) ≥ 90 mmHg. H-type hypertension is defined as three measurements of systolic blood pressure (SBP) ≥ 140 mmHg and/or diastolic blood pressure (DBP) ≥ 90 mmHg and a plasma Hcy level ≥ 15 µmol/L.

### Data collection and measurement

This study is from the project “Survey on the Current Situation of Chronic and Endemic Diseases and Their Influencing Factors among Tibetans in Tibetan Areas”. Questionnaires were used to collect basic personal information such as age and gender.

Blood pressure was measured with calibrated electronic sphygmomanometer after at least 5-min while seated, using standardised procedures. We used the mean of three pressure readings.

A total of 5mL fasting venous blood of the subjects was collected and sent to the laboratory department of Tibetan Hospital of Tibet Autonomous Region for biochemical tests. The analysis included the determination of plasma Hcy levels, blood lipid parameters, renal function markers, blood uric acid concentration, as well as other pertinent indices.

Oral mucosal cells were collected from the investigated subjects using an oral mucosal cell sampler for subsequent gene detection. After collection, the specimens were stored in a refrigerator at -20 °C and sent to Chengdu Yishan Medical Laboratory within 7 days for testing. The gene detection process involved routine PCR amplification was performed using the MTHFR C677T gene detection kit. High-quality genomic DNA was extracted from oral swabs using a highly efficient genomic DNA extraction kit. Subsequently, primers and probes were synthesized and debugged; then the MTHFR C677T locus was detected using the fluorescent probe-based method. Ultimately, the results were output and analyzed.

### Quality control

In order to ensure the quality of questionnaire, the survey process will be conducted by the unified training Tibetan-Chinese bilingual investigators with conducted face-to-face information inquiry and collection, and filled in the questionnaire truthfully.

The blood biochemical tests in this study were conducted at the Department of Laboratory of Tibetan Hospital of Tibet Autonomous Region, while the genetic tests were performed at the Institute of Chengdu Yishan Medical Laboratory. All the aforementioned testing institutions adhered to the national qualification standards for testing and implemented regular quality control measures for both biochemical and genetic analyses.

### Statistical analysis

The data were subjected to statistical analysis using SPSS 26.0. The measurement data that adhered to a normal distribution were represented as mean ± standard deviation (x̅±SD), with the comparison between two groups and multiple groups, T-test, F-test, and one-way analysis of variance (ANOVA) were utilized, respectively. The statistical data were expressed in terms of rates and composition ratios, with the comparison between groups conducted through the *χ*^*2*^ test. The distribution of gene frequencies was tested by Hardy-Weinberg genetic equilibrium test.

Logistic regression was used to assess influencing factors related to H-type hypertension, followed by the construction of a nomogram prediction model for H-type hypertension using R software version 4.3.1. The model’s goodness of fit was evaluated with the Hosmer-Lemeshow test, and its performance was further assessed through ROC curve analysis. A Calibration curve was generated through 1000 Bootstrap self-sampling iterations. In the Hosmer-Lemeshow goodness-of-fit curve, if *p* > 0.05, it indicates good accuracy of the predictive model. The discriminative ability is assessed using Harrell’s C statistical concordance index (C-index). Typically, if the C-index is > 0.7, it is considered indicative of reasonable discrimination. An area under the ROC curve > 0.75 suggests strong discrimination.A prediction model is considered more accurate when the calibration curve closely aligns with the standard curve. Two-tailed *p* < 0.05 were considered to indicate statistical significance.

## Results

### Characteristics of the study population

Patients with H-type hypertension accounted for 21.14% and 84.31% of the total population and hypertensive population. The four groups showed statistically significant differences in age, gender, systolic blood pressure(SBP), diastolic blood pressure(DBP), Hcy, fasting plasma glucose(FPG), total cholesterol(TC), triglycerides, HDL cholesterol(HDL-C), LDL cholesterol(LDL-C), urea nitrogen(UREA), creatinine(CREA), and uric acid(UA)(all *p* < 0.05). In the H-type hypertension group, the levels of age, DBP, Hcy, FPG, TC, TG, LDL-C, CREA, and UA are all significantly higher compared to the other three groups, with statistical significance (all *p* < 0.05) (Table 1).

**Table 1:** General demographic characteristics

Variables	Control	Isolated Hyperhomocysteinemia	Essential hypertension	H-type hypertension	F/χ^2^	*p*-value
**Gender** (M/F)	419 (96/323)	1314 (584/730)^a^	91 (18/73)^b^	489 (242/247)^a c^	94.758	< 0.001
**Age** (years)	39.38 ± 11.28	42.01 ± 13.60^a^	48.90 ± 12.73^a b^	54.99 ± 12.07^a b c^	150.581	< 0.001
**Smoking**	26	155^a^	7	47^a^	11.801	0.008
**Drinking**	53	209	17	89	5.770	0.123
**SBP** (mm Hg)	111.89 ± 12.06	114.26 ± 11.04^a^	148.40 ± 51.35^a b^	148.19 ± 20.95^a b^	595.561	< 0.001
**DBP** (mm Hg)	73.84 ± 8.58	75.94 ± 8.18^a^	95.43 ± 8.56^a b^	99.04 ± 10.73^a b c^	989.013	< 0.001
**Hcy** (µmol/L)	12.10 ± 2.40	23.63 ± 9.91^a^	12.24 ± 2.71^b^	24.50 ± 9.48^a c^	240.827	< 0.001
**FPG** (mmol/L)	4.61 ± 0.83	4.74 ± 0.97^a^	4.85 ± 1.49	5.03 ± 1.17^a b^	14.481	< 0.001
**TC** (mmol/L)	4.65 ± 1.39	6.20 ± 1.65^a^	5.05 ± 1.41^b^	6.47 ± 1.74^a b c^	127.210	< 0.001
**TG** (mmol/L)	0.85 ± 0.55	1.28 ± 0.83^a^	1.08 ± 0.64^a b^	1.51 ± 0.88^a b c^	54.565	< 0.001
**HDL-C** (mmol/L)	1.51 ± 0.58	1.83 ± 0.64^a^	1.50 ± 0.52^b^	1.75 ± 0.59^a c^	35.055	< 0.001
**LDL-C** (mmol/L)	2.28 ± 0.78	3.20 ± 1.00^a^	2.62 ± 0.86^a b^	3.49 ± 1.12^a b c^	132.913	< 0.001
**UREA** (mmol/L)	5.04 ± 1.55	6.45 ± 2.14^a^	4.63 ± 1.75^b^	6.25 ± 2.37^ac^	64.450	< 0.001
**CREA** (µmol/L)	48.96 ± 15.30	71.07 ± 35.63^a^	48.97 ± 16.89^b^	71.24 ± 26.61^a c^	70.292	< 0.001
**UA** (µmol/L)	281.96 ± 97.01	421.94 ± 127.99^a^	301.35 ± 104.55^b^	443.83 ± 140.46^a b c^	176.479	< 0.001

*Note : SBP* Systolic blood pressure, *DBP* Diastolic blood pressure, *Hcy* Homocysteine, *FPG* Fasting plasma glucose, *TC* Total cholesterol, *TG* Triglycerides, *HDL-C* High-density lipoprotein cholesterol, *LDL-C* Low-density lipoprotein cholesterol, *UREA* Urea nitrogen, *CREA* Creatinine, *UA* Uric acid.1 mmHg = 0.133 kPa. ^a^*p*<0.05 vs. Control group, ^b^*p*<0.05 vs. isolated Hyperhomocysteinemia group,^*c*^*p*<0.05 vs. essential hypertension group

### Hardy-Weinberg genetic equilibrium test

The results of genetic balance test showed that the distribution of MTHFR C677T genotype in the four groups was in genetic balance (H-type hypertension group, *χ*^*2*^ = 2.089, *p* = 0.352; essential hypertension group, *χ*^*2*^ = 0.288, *p* = 0.866; isolated Hyperhomocysteinemia group, *χ*^*2*^ = 0.380, *p* = 0.827; control group, *χ*^*2*^ = 1.612, *p* = 0.447), indicating that the enrolled subjects were genetically, representative of the Tibetan population in Tibet.

### Distribution of MTHFR C677T genotypes and alleles

There were statistically significant differences in the distribution frequencies of CC, CT, and TT genotypes, recessive model CC + CT, and TT genotypes, and C and T alleles between the four groups (all *p* < 0.05). The distribution frequencies of the CC, CT, and TT genotypes of MTHFR C677T in the H-type hypertension group were statistically significant (all *p* < 0.05) when compared to the other three groups. However, the distribution frequencies of the C and T alleles in the H-type hypertension group did not show statistical significance when compared to the other three groups (all *p* > 0.05). Compared to the control group, there was a statistically significant difference in the distribution frequencies of the recessive genotype in both the H-type hypertension group and the isolated Hyperhomocysteinemia group (all *p* < 0.05) (Table 2).

**Table 2:** Polymorphism, recessive and allele frequencies of MTHFR C677T gene [n (%)]

Groups	Genotype			Recessive model genotype		Allele	
	CC	CT	TT	CC + CT	TT	C	T
**Control**	273 (65.16)	137 (32.69)	9 (2.15)	410 (97.85)	9 (2.15)	683 (81.50)	155 (18.50)
**Isolated** **Hyperhomocy-** **steinemia**	747 (56.85)^a^	495 (37.67)^a^	72 (5.48)^a^	1242 (94.52)^a^	72 (5.48)^a^	1989 (75.68)^a^	639 (24.32)^a^
**Essential hypertension**	59 (64.84)^b^	32 (35.16)^b^	0 (0.00)^b^	91 (100.00)^b^	0 (0.00)^b^	150 (82.42)^b^	32 (17.58)^b^
**H-type** **hypertension**	307 (62.78)^abc^	152 (31.08)^abc^	30 (6.14)^abc^	459 (93.87)^a^	30 (6.13)^a^	766 (78.32)	212 (21.68)
***χ*** ^***2***^	23.972			14.275		15.592	
***p-value***	0.001			0.003		0.001	

*Note: *^a^*p*<0.05 vs. Control group, ^b^*p*<0.05 vs. isolated Hyperhomocysteinemia group, ^c^*p* < 0.05 vs. essential hypertension group

### Association of different genotypes of MTHFR C677T with Hcy and lipids in H-type hypertension population

In the H-type hypertension group, the Hcy levels for the MTHFR C677T genotypes CC, CT, and TT were (23.28 ± 7.59), (24.72 ± 9.20), and (35.84 ± 17.72) µmol/L, respectively. There was a statistically significant difference among the genotypes (*F* = 26.593, *p* < 0.001). Specifically, the TT genotype exhibited the highest Hcy levels, followed by CT, with significant differences noted in comparisons between TT and CT (*p* < 0.001), TT and CC (*p* < 0.001), while the comparison between CT and CC did not yield statistical significance (*p* = 0.107). Among the lipid components, only low-density lipoprotein cholesterol (LDL-C) showed a statistically significant difference between the 3 groups of genotypes (F = 3.065, *P* < 0.05) (Table 3).

**Table 3:** Association of different genotypes of MTHFR C677T with Hcy and lipids in H-type hypertension population

Variables	CC mould	CT mould	TT mould	F	*P* value
**Hcy** (µmol/L)	23.28 ± 7.59	24.72 ± 9.20	35.84 ± 17.72^ab^	26.593	< 0.001
**TC** (mmol/L)	6.46 ± 1.70	6.35 ± 1.80	7.20 ± 1.71	2.970	0.052
**TG** (mmol/L)	1.50 ± 0.90	1.47 ± 0.81	1.81 ± 1.06	1.942	0.144
**HDL-C** (mmol/L)	1.73 ± 0.59	1.77 ± 0.57	1.89 ± 0.63	0.949	0.388
**LDL-C** (mmol/L)	3.50 ± 1.08	3.38 ± 1.18	3.93 ± 1.11^b^	3.065	0.048

*Note: *^a^*p*<0.05 vs. CC genotype, ^b^*p*<0.05 vs. CT genotype

### Logistic regression analysis of factors related to H-type hypertension

Using H-type hypertension status as the dependent variable, a logistic regression analysis was conducted with age, gender, smoking, FPG, TC, TG, HDL-C, LDL-C, UA, CREA, UREA and MTHFR C677T genotype as independent variables. The results showed that age, Urea, UA, TC, TG, LDL-C and MTHFR C677T TT genotype were related to Tibetan H type hypertension (*p* < 0.05).Age, UA, TG, LDL-C were risk factors for the prevalence of H-type hypertension, the OR (95% CI) was 1.083(1.073–1.094), 1.002(1.001–1.004), 1.240(1.050–1.464) and 2.274(1.432–3.611),respectively. MTHFR C677T TT genotype patients with H-type hypertension OR (95% CI) was 1.629(1.004–2.643) (Table 4).

**Table 4:** Multivariate logistic regression analysis of the influencing factors of H-type hypertension

Variables		β	SE	Wald	*p*-value	OR(95%CI)
	**Age**	0.080	0.005	273.319	< 0.001	1.083(1.073–1.094)
	**Gender**	-0.031	0.141	0.049	0.825	0.969(0.735–1.278)
	**Smoking**	0.044	0.203	0.048	0.826	1.046(0.702–1.556)
	**FPG**	0.013	0.054	0.058	0.810	1.013(0.911–1.126)
	**UREA**	-0.075	0.030	6.292	0.012	0.928(0.875–0.984)
	**CREA**	0.002	0.002	0.580	0.446	1.002(0.998–1.006)
	**UA**	0.002	0.001	16.205	< 0.001	1.002(1.001–1.004)
	**TC**	-0.453	0.179	6.396	0.011	0.636(0.448–0.903)
	**TG**	0.215	0.085	6.413	0.011	1.240(1.050–1.464)
	**HDL-C**	0.242	0.200	1.465	0.226	1.273(0.861–1.883)
	**LDL-C**	0.821	0.236	12.108	0.001	2.274(1.432–3.611)
	**MTHFR C677T** **TT genotype**	0.488	0.247	3.913	0.048	1.629(1.004–2.643)

*Note*: *FPG* Fasting plasma glucose, *UREA* Urea nitrogen, *CREA* Creatinine, *UA* Uric acid, *TC* Total cholesterol, *TG* Triglycerides, *HDL-C* High-density lipoprotein cholesterol, *LDL-C* Low-density lipoprotein cholesterol

Assignments, dependent variable, H-type hypertension(No = 0,Yes = 1); independent variables, Gender (Male = 0,Female = 1); Smoking(No = 0,Yes = 1); MTHFR genotypes (CC + CT = 0,TT = 1) and age, FPG, UREA, CREA, UA, TC, TG, LDL-C and HDL-C were continuous variables for the analysis

### Nomogram construction and validation

**Fig. 1:**
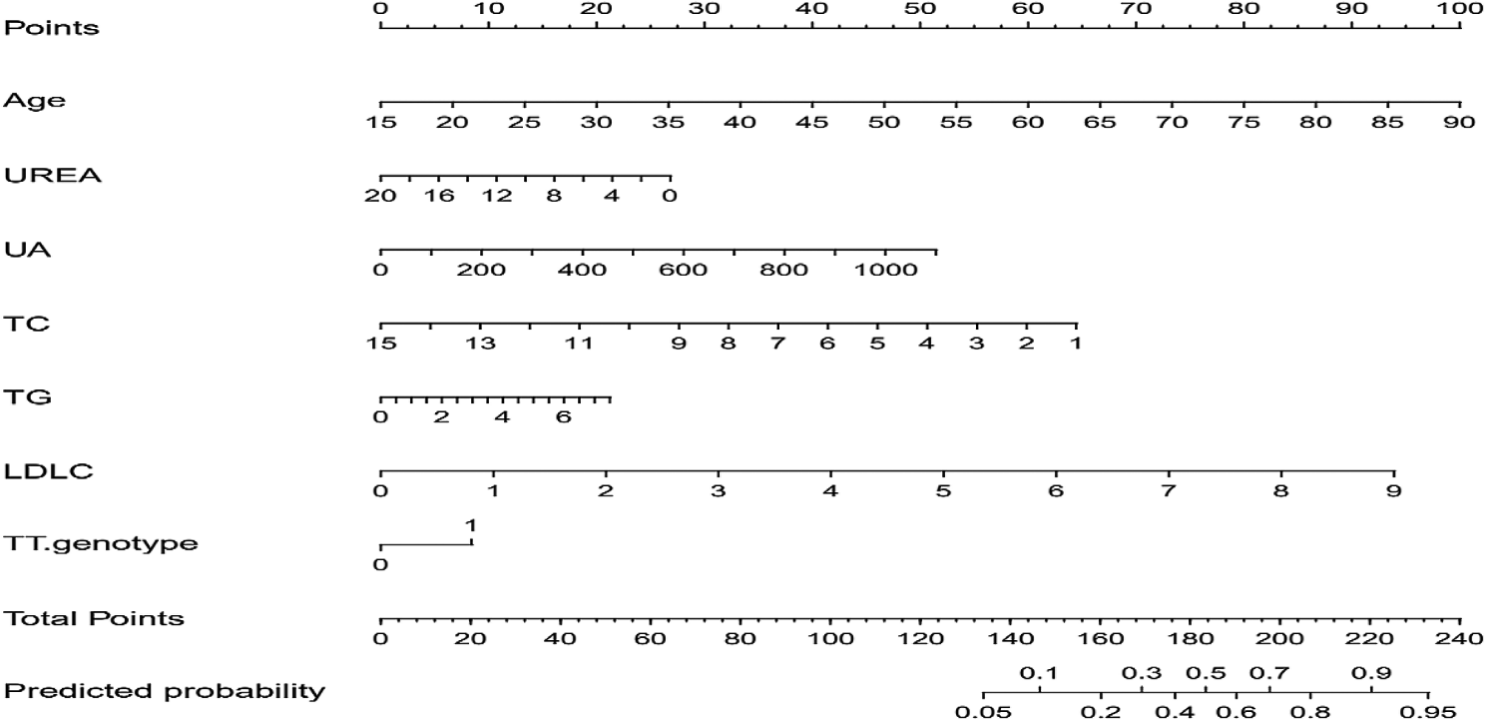
Nomogram for predicting the probability of H-type hypertension

To construct a nomogram based on the logistic regression results, which includes seven factors: age, UREA, UA, TC, TG, LDL-C, and MTHFR C677T TT genotype. This model can calculate scores for each independent factor and aggregate a total score, where the total score corresponds to the predicted probability of developing H-type hypertension. The model’s results indicate that for every five years of age, the score increases by 6 points; for a 200µmol/L increase in UA, the score increases by 9 points; for a 2mmol/L increase in TG, the score increases by 5.5 points; for a 1mmol/L increase in LDLC, the score increases by 10 points; and individuals with the TT genotype receive 8 points (Fig. 1). The higher the total score, the greater the risk of developing H-type hypertension.

**Fig. 2:**
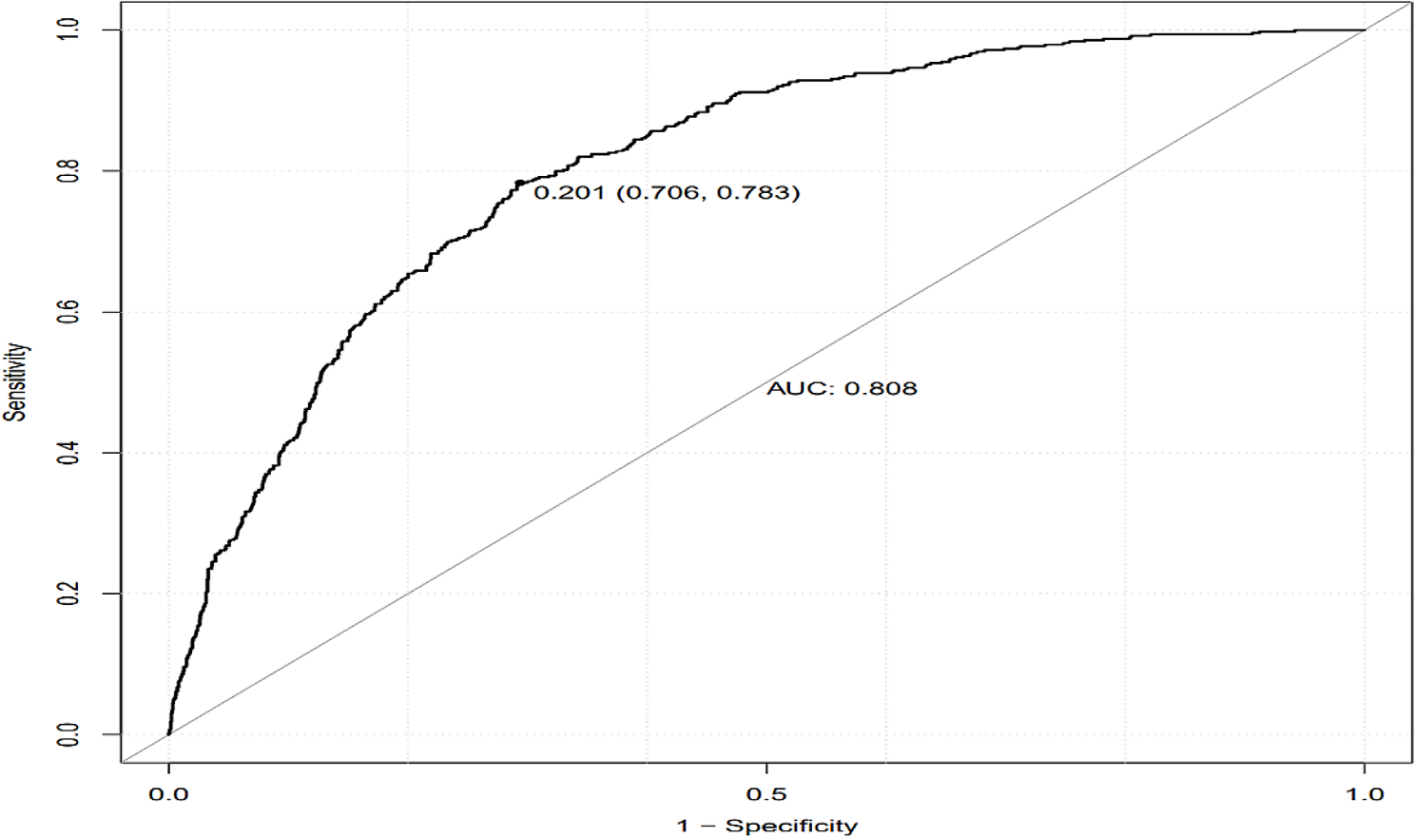
ROC curve of the nomogram model

**Fig. 3:**
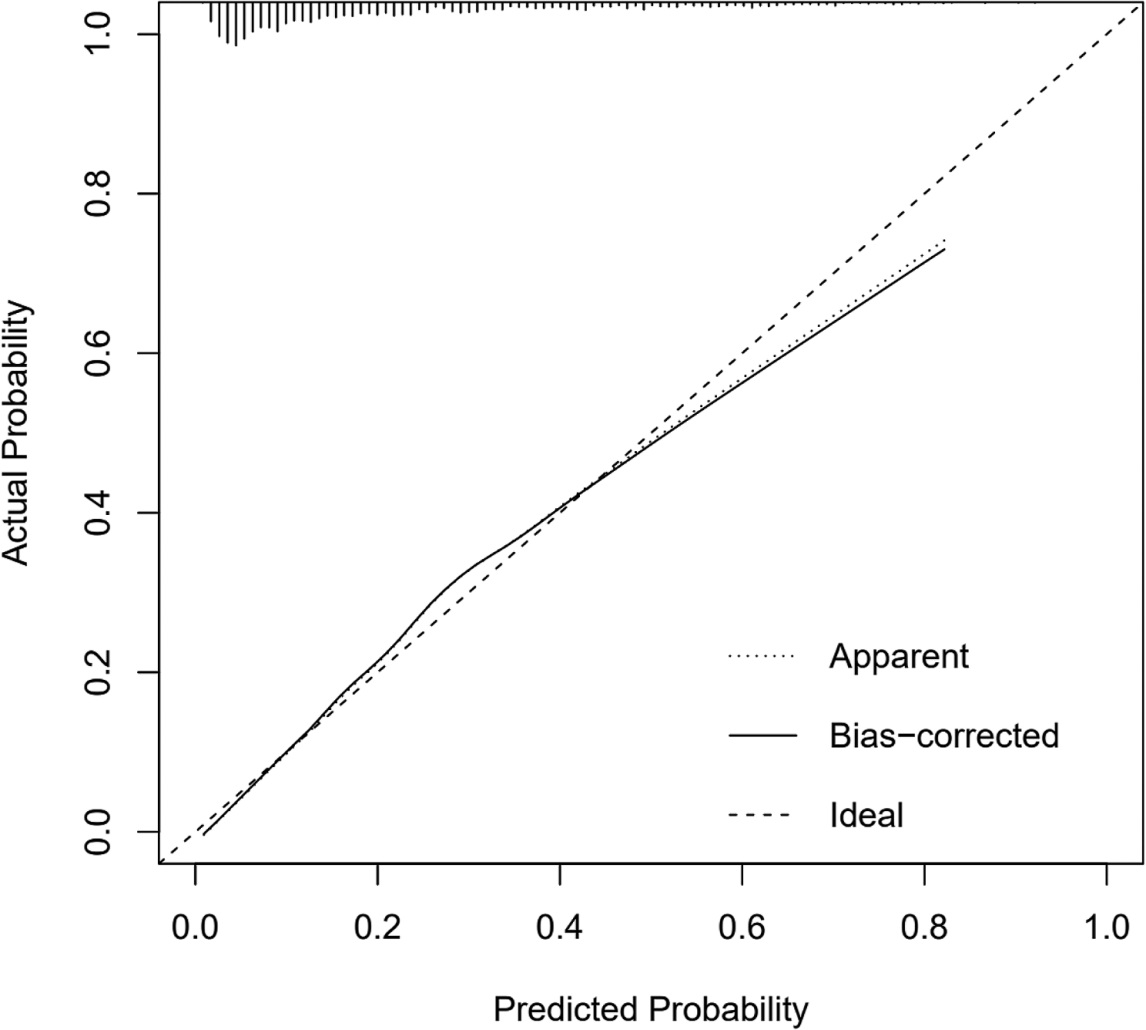
Calibration curves

By calculating the C-index of this model (equivalent to the AUC under the ROC curve) as 0.808 (95% CI: 0.788–0.828), it suggests that the model exhibits a relatively strong discriminatory ability (Fig. 2).The calibration curve shows that the calibration curve is basically close to the predicted curve and the ideal curve, with the Brier score = 0.132 and the Mean absolute error = 0.013,indicates a satisfactory level of accuracy in the model’s predictions (Fig. 3).Furthermore, the Hosmer-Lemeshow (H-L) fit curve was *χ*^*2*^ *=* 11.848, *p* = 0.158 > 0.05, the difference was not statistically significant, indicating that the model had a strong explanatory power and a good degree of fit.

## Discussion

This study showed that the prevalence of H-type hypertension in Tibetan areas among the Tibetan population has reached a notable prevalence of 21.14%, surpassing the previous estimate of 15.1% among Tibetans residing in high-altitude regions [17]. Patients with H-type hypertension accounted for 84.31% of the total hypertensive population, higher than the baseline data of H-type hypertension in 79.68% of the hypertensive population in China from the Essential Stroke Prevention Study [18]. These results may be related to the local population’s exposure to low-pressure, low-oxygen environments, high salt and high-fat diets, and low intake of fresh fruits and vegetables.The H-type hypertension group exhibits a higher average age, with elevated levels of Hcy, TC, TG, LDL-C, UA and CREA compared to the other three groups. This may be due to a decrease in digestive and absorptive capacity with age, as well as impaired hepatic and renal metabolic function, resulting in decreased Hcy clearance. Furthermore, a high-sodium diet may impede the absorption of folate and B-complex vitamins, resulting in an abnormal elevation of plasma Hcy levels, thereby promoting the occurrence of H-type hypertension [19, 20]. High plasma Hcy interacts with disorders of lipid metabolism and may play a synergistic and facilitative role in causing vascular endothelial damage and subsequent hypertension [21].

Our study showed that the frequencies of CC, CT, and TT genotypes in the MTHFR C677T gene were 62.78%, 31.08%, and 6.14%, and the frequencies of C and T alleles were 78.32% and 21.68%, in Tibetan patients with H-type hypertension located in the southwest, and the CC genotype and C allele had the highest distribution frequency. However, the frequencies of the three genotypes were 13.51%, 45.94%, and 50.45%, and the frequencies of the C and T alleles were 44.3% and 55.7%, among the Qinghai Han people located in the northwest region, and the TT genotype and T allele had the highest distribution frequency [22]. In Guangxi Zhuang people, which is located in South China, the frequencies of the three genotypes were 67.1%, 25%, and 7.89%, and the frequencies of C and T alleles were 40.74% and 42.47%, with the highest frequency of CC genotype and T allele distribution [23]. This may suggest that there is uncertainty in the distribution of the C677T gene variant among Tibetan H-type hypertension patients, with gene mutations exhibiting regional and ethnic specificity. Joint comparison of the relationship between different genotypes of MTHFR C677T and Hcy levels in the H-type hypertension population showed that Hcy levels were much higher in the TT group than in the CT and TT groups. It was further hypothesized that the TT genotype may be responsible for the development of H-type hypertension and for the worsening of H-type hypertension due to elevated Hcy levels.Among the lipid components, only low-density lipoprotein cholesterol (LDL-C) showed a statistically significant difference between the 3 groups of genotypes. It has been suggested that the association between MTHFR C677T and lipids is inconclusive and may be related to genetic heterogeneity between different regions or ethnic groups [24].

Logistic regression analysis showed that the MTHFR gene C677T TT genotype had an increased risk of developing H-type hypertension (OR = 1.692). At the same time, logistic regression results also mentioned that age, UA, TG, LDL-C were the risk factors of H-type hypertension. This is generally consistent with previous research findings, and if there are variations in influencing factors, they should be considered from the perspective of individual pathophysiology and lifestyle [25, 26]. The study suggests that TG and LDL-C are independent variables influencing disease progression [27]. Meta-analysis results indicate that in the overall genetic model of the human population, individuals carrying the TT genotype have an increased risk of developing H-type hypertension [28]. Most studies rely on single logistic regression analysis, which has limitations in interpreting the results. Therefore, combining the construction of a risk prediction model tailored to the local population is of significant practical importance in preventing and controlling the development of H-type hypertension.Based on the results of multifactor logistic regression analysis, this study has developed a nomogram prediction model for H-type hypertension, incorporating seven factors. The model showed that the score increased by 6 points for every 5-year increase in age; the score increased by 9 points for every 200 umol/L increase in UA; the score increased by 5.5 points for every 2-mmol/L increase in TG; the score increased by 10 points for every 1-mmol/L increase in LDLC; and individuals with the TT genotype received a score of 8 points. The higher the total score, the greater the risk of developing type h hypertension. Abnormal increases in the above indicators lead to varying degrees of risk for the development of H-type hypertension; therefore, it is recommended that the above indicators be included in the monitoring and management of H-type hypertension in the daily management of H-type hypertension.The model’s accuracy and discriminative ability were assessed using the H-L goodness-of-fit curve, AUC, and C-index index. The results indicate that this model possesses a high degree of discrimination and accuracy, making it a valuable tool for healthcare professionals from various fields to preliminarily assess individual disease risk and provide targeted prevention and treatment measures. Studies have demonstrated the efficacy of regular aerobic exercise in stabilizing blood pressure in individuals diagnosed with H-type hypertension [29]. This exercise program not only enhances cardiorespiratory endurance, but contributes to the improvement and management of the patient’s pathological state. Aerobic exercise may be considered as a treatment and management approach for the H-hypertensive population.

The participants in this study were Tibetan people in Tibet who have been living in a high-altitude environment at an average altitude of 4200 m. Further in-depth studies are needed to determine whether the group’s ability to adapt to high altitude, low pressure and low oxygen leads to a different genetic basis and whether the unique Tibetan dietary pattern of high salt and high fat, and low intake of fresh fruits and vegetables due to the survival environment causes differences in the prevalence of H-type hypertension.

## Conclusion

The mutation occurring at the MTHFR C677T TT locus is a potential contributing factor to the development of H-type hypertension in the Tibetan population residing in Tibet. Additionally, age, UA, TG, LDL-C and the TT genotype are considered as risk factors for the onset of H-type hypertension in this population. Therefore, it is advocated that hypertensive patients should, in addition to routine blood pressure measurements, regularly monitor their homocysteine (Hcy) levels to prevent the synergistic effects between the two, and implement targeted preventive and control measures. These measures include strengthening health education in agricultural and pastoral regions, improving dietary patterns, promoting scientifically validated aerobic exercise, and incorporating rational interventions such as adequate folate intake.All of these efforts aim to improve the occurrence of adverse cardiovascular and cerebrovascular events.

### Limitations

Our study has limitations due to its cross-sectional design, which precludes establishing causal relationships between risk factors and the occurrence of H-type hypertension. In terms of model validation, we only conducted internal validation and lack external validation to assess the model’s generalizability. Additionally, there is currently limited research on the genotypes of H-type hypertension patients in the Tibetan region. Therefore, it is necessary to conduct prospective multicenter cohort studies with larger sample sizes, encompassing various altitudes and geographical regions, to explore the relationship between genetic factors and H-type hypertension in the Tibetan population.

## Data Availability

The datasets used during the current study are available from the corresponding author on reasonable request.

## Abbreviations

Hcy, homocysteinemia: MTHFR,5,10-methylenetetrahydrofolate reductase
SBP: Systolic blood pressure
DBP: Diastolic blood pressure
ANOVA: One-way analysis of variance
C-index: Harrell’s C statistical consistency index
FPG: Fasting plasma glucose;
TC: Total cholesterol
HDL-C: High-density lipoprotein cholesterol; LDL-C, low-density lipoprotein cholesterol
UREA: Urea nitrogen;
CREA: Creatinine; UA, Uric acid
OR: Odds Ratio;

## References

[1] Liang Z, Fan FF, Zhang Y, Qin XH, Li JP, Huo Y (2022). [Rate and characteristics of H-type hypertension in Chinese hypertensive population and comparison with American population]. Beijing Da Xue Xue Bao.

[2] Moretti R, Caruso P (2019). The controversial role of Homocysteine in Neurology: from labs to clinical practice. Int J Mol Sci.

[3] Clarke R, Bennett DA, Parish S, Verhoef P, Dötsch-Klerk M, Lathrop M (2012). Homocysteine and Coronary Heart Disease: Meta-analysis of MTHFR Case-Control studies, avoiding publication Bias. PLoS Med.

[4] Liu S, Liu M, Li Q, Liu X, Wang Y, Mambiya M (2019). Association of single nucleotide polymorphisms of MTHFR, TCN2, RNF213 with susceptibility to hypertension and blood pressure. Biosci Rep.

[5] Zhang C, Li J, Zhou J, Zheng Q, Dong R, Xing E (2022). Effect of MTHFRC677 T gene polymorphism on early morning blood pressure in Elderly Female patients with H-Type hypertension. Contrast Media Mol Imaging.

[6] Frosst P, Blom HJ, Milos R, Goyette P, Sheppard CA, Matthews RG (1995). A candidate genetic risk factor for vascular disease: a common mutation in methylenetetrahydrofolate reductase. Nat Genet.

[7] Holmes MV, Newcombe P, Hubacek JA, Sofat R, Ricketts SL, Cooper J (2011). Effect modification by population dietary folate on the association between MTHFR genotype, homocysteine, and stroke risk: a meta-analysis of genetic studies and randomised trials. Lancet.

[8] He Q, Wei Y, Zhu H, Liang Q, Chen P, Li S (2024). The combined effect of MTHFR C677T and A1298C polymorphisms on the risk of digestive system cancer among a hypertensive population. Discov Oncol.

[9] Yang B, Fan S, Zhi X, Xia R, Wang Y, Zheng Q (2017). Geographical and ethnic distribution of MTHFR gene polymorphisms and their associations with diseases among Chinese population. Clin Genet.

[10] Zhang C, Xin Q-P, Xie Y-B, Guo X-Y, Xing E-H, Dou Z-J (2024). Relationship between methylenetetrahydrofolate reductase C677T gene polymorphism and neutrophil gelatinase-associated lipocalin in early renal injury in H-type hypertension. BMC Cardiovasc Disord.

[11] Raghubeer S, Matsha TE (2021). Methylenetetrahydrofolate (MTHFR), the one-Carbon cycle, and Cardiovascular risks. Nutrients.

[12] Petrone I, Bernardo PS, dos Santos EC, Abdelhay E (2021). MTHFR C677T and A1298C polymorphisms in breast Cancer, Gliomas and gastric Cancer: a review. Genes.

[13] Zhang Y-X, Yang L-P, Gai C, Cheng C-C, Guo Z, Sun H-M (2022). Association between variants of MTHFR genes and psychiatric disorders: a meta-analysis. Front Psychiatry.

[14] Jouibari RM, Movafagh A, Molaei A (2021). Association between maternal and fetal MTHFR C677T and MTRR A66G polymorphisms with the risk of NTDs: a systematic review and Meta-analysis study. Iran Red Crescent Med J.

[15] Yao C-Y, Lu H-Z, Zhang L-W,Song J-X, He H-X, Zhang R-X et al. [The polymorphism of methylenetetrahydrofolate reductase gene C677Tin Han population in Qinhuangdao]. Chin Prev Med. 2020:21;490–494. 10.16506/j.1009-6639.2020.05.003.

[16] 2018 Chinese Guidelines for Prevention and Treatment of Hypertension—A report of the Revision Committee of Chinese Guidelines for Prevention and Treatment of Hypertension (2019). J Geriatr Cardiol JGC.

[17] Guan H-Y. [Investigation on homocysteine level and its related gene polymorphism of hypertension in native Tibetans]master.Tibet University,2021. 10.27735/d.cnki.gx-zdx.2021.000148.

[18] Qin X, Li Y, Sun N, Wang H, Zhang Y, Wang J (2017). Elevated homocysteine concentrations decrease the Antihypertensive Effect of Angiotensin-converting enzyme inhibitors in Hy-pertensive patients. Arterioscler Thromb Vasc Biol.

[19] Shi X-J, Duan K-D,Xiao (2023). Y.[Influence factor analysis of hypertension complicated with hype-rhomocysteinemia]. J Huaihai Med.

[20] Xu R, Huang F, Wang Y, Liu Q, Lv Y, Zhang Q (2020). Gender- and age-related differences in homocysteine concentration: a cross-sectional study of the general population of China. Sci Rep.

[21] Liu Y, Xu C, Wang Y, Yang C, Pu G, Zhang L (2023). Association analysis of MTHFR (rs1801133 and rs1801131) and MTRR (rs1801394) gene polymorphisms towards the develo-pment of hypertension in the Bai population from Yunnan. China Clin Exp Hypertens.

[22] Chen L. [Association between the polymorphism of MTHFR C677T and H type hypertension in the Han population live in Qinghai Province,China] master. Ningxia Medical University; 2016.

[23] Huang LQ, Wu CX, Wei HQ, Xu G (2020). Clinical characteristics of H-type hypertension and its relationship with the MTHFR C677T polymorphism in a Zhuang population from Guangxi, China. J Clin Lab Anal.

[24] Li Q, Sun XL, Song B, Lyu CX, Feng XQ (2022). [Association of small dense low-density lipoprotein cholesterol with H-type hypertension and MTHFR gene polymorphism]. Zhonghua Yu Fang Yi Xue Za Zhi.

[25] Qian X, Cao H, Zhang J, Gu Z, Tang W, Shen L (2021). The prevalence, relative risk factors and MTHFR C677T genotype of H type hypertension of the elderly hypertensives in Shanghai, China: a cross-section study. BMC Cardiovasc Disord.

[26] Analysis of metabolism-related indicators. and MTHFR gene polymorphism in patients with H-type hypertension - Minerva Medica 2017 April;108(2):103-7 n.d. https://www.minervamed-ica.it/en/journals/minerva-medica/article.php?cod=R10Y2017N02A0103 (accessed November 7, 2023). 10.23736/S0026-4806.16.04951-X 27973469

[27] Yuan W, Shao Y, Zhao D, Zhang B (2023). Correlation analysis of lipid accumulation index, triglyceride-glucose index and H-type hypertension and coronary artery disease. PeerJ.

[28] Liao S, Guo S, Ma R, He J, Yan Y, Zhang X (2022). Association between methylenetetrahydrofolate reductase (MTHFR) C677T polymorphism and H-type hypertension: a systematic review and meta‐analysis. Ann Hum Genet.

[29] Miao W. Effects of regular aerobic exercise on cardiorespiratory endurance and blood pressure responses to exercise in patients with h-type hypertension. Acta Med Mediterr 2021:2541–6. 10.19193/0393-6384_2021_4_394.

